# Evidence for widespread changes in promoter methylation profile in human placenta in response to increasing gestational age and environmental/stochastic factors

**DOI:** 10.1186/1471-2164-12-529

**Published:** 2011-10-28

**Authors:** Boris Novakovic, Ryan K Yuen, Lavinia Gordon, Maria S Penaherrera, Andrew Sharkey, Ashley Moffett, Jeffrey M Craig, Wendy P Robinson, Richard Saffery

**Affiliations:** 1 Cancer, Disease and Developmental Epigenetics, Murdoch Childrens Research Institute, Royal Children's Hospital and Department of Paediatrics, University of Melbourne, Parkville, Victoria 3052, Australia; 2 Early Life Epigenetics, Murdoch Childrens Research Institute, Royal Children's Hospital and Department of Paediatrics, University of Melbourne, Parkville, Victoria 3052, Australia; 3 Department of Medical Genetics, University of British Columbia, Child & Family Research Institute, 950 West 28th Ave., Vancouver, BC, Canada; 4 Bioinformatics Unit, Murdoch Children's Research Institute, Royal Children's Hospital, Flemington Road, Parkville, Victoria 3052, Australia; 5 Department of Pathology, University of Cambridge, Cambridge, CB2 1QP, UK

## Abstract

**Background:**

The human placenta facilitates the exchange of nutrients, gas and waste between the fetal and maternal circulations. It also protects the fetus from the maternal immune response. Due to its role at the feto-maternal interface, the placenta is subject to many environmental exposures that can potentially alter its epigenetic profile. Previous studies have reported gene expression differences in placenta over gestation, as well as inter-individual variation in expression of some genes. However, the factors contributing to this variation in gene expression remain poorly understood.

**Results:**

In this study, we performed a genome-wide DNA methylation analysis of gene promoters in placenta tissue from three pregnancy trimesters. We identified large-scale differences in DNA methylation levels between first, second and third trimesters, with an overall progressive increase in average methylation from first to third trimester. The most differentially methylated genes included many immune regulators, reflecting the change in placental immuno-modulation as pregnancy progresses. We also detected increased inter-individual variation in the third trimester relative to first and second, supporting an accumulation of environmentally induced (or stochastic) changes in DNA methylation pattern. These highly variable genes were enriched for those involved in amino acid and other metabolic pathways, potentially reflecting the adaptation of the human placenta to different environments.

**Conclusions:**

The identification of cellular pathways subject to drift in response to environmental influences provide a basis for future studies examining the role of specific environmental factors on DNA methylation pattern and placenta-associated adverse pregnancy outcomes.

## Background

The human placenta is a temporary organ that facilitates the exchange of nutrients, gas and waste between maternal and fetal circulations. In order to carry out these functions, it is comprised of heterogeneous cell types including several trophoblast cell populations (cytotrophoblasts, extra-villous trophoblasts and syncytiotrophoblast), fibroblasts, mesenchymal cells, as well as fetal and maternal vascular tissue and blood cells. The extra-villous trophoblast cells must first invade the maternal decidua and remodel maternal arteries, to allow direct contact between maternal blood and the placental syncytiotrophoblast cell layer [1]. In addition to these traditional roles, the placenta is also important in shielding the developing fetus from the maternal immune system [2].

The placenta also undergoes several physiological changes throughout gestation, with one of the most significant being the flooding of placenta villi by maternal blood at the end of the first trimester (~12 weeks gestation), resulting in a rise in oxygen concentration as well as a decrease in trophoblast invasion. It is believed that the inability of the placenta to respond to this change in oxygen concentration can lead to placental disease, such as preeclampsia [3,4].

The molecular mechanisms behind these morphological and functional changes are now beginning to be understood at both the gene-specific and genome-wide level. Wide-ranging genome-wide gene expression differences between placentas at different gestational ages were reported in two recent studies [5,6]. Despite sampling from different locations within the placenta, many changes were found in common between the two studies, each of which reported changes in expression with increasing gestational age in genes involved in cell cycle and immune response. This suggests that gene expression changes are needed for physiological needs of the developing placenta, such as shielding the fetus from the maternal immune system [2]. Genes involved in Wnt signalling also showed expression changes over time [5,6] that resulted in decreasing levels of β-catenin later in gestation, possibly linked to decreasing placental invasiveness [6].

The importance of epigenetic factors in placental development and function has long been known through the study of imprinted genes [7,8] and it is increasingly clear that the placenta displays a unique epigenetic profile. However, the extent to which epigenetic modifications, specifically DNA methylation, contribute to placental function have only recently been widely examined (reviewed in [9].

Due to its role as the interface between the mother and fetus, the placenta is exposed to a myriad of environmental factors, some of which have been shown to alter placental gene expression, as well as epigenetic marks [10]. These include diet [11,12], smoking [13], and assisted reproductive techniques [14,15]. Mounting evidence implicates epigenetic marks, such as DNA methylation, in mediating environmentally-induced regulation of genome function. More studies into the effects of the environment on the placental epigenome are warranted due the importance of this organ in regulating pregnancy development.

Several genome-scale DNA methylation studies have focused on finding tissue-specific differentially methylated regions (tDMRs) between placenta and maternal blood, as a means of detecting placental pathologies and fetal chromosomal trisomies using non-invasive methods (reviewed in [16-19]. This strategy has recently resulted in the development of the first non-invasive blood test for Down syndrome [20]. However, we, and others have revealed substantial inter-individual DNA variation in placental methylation profile [21,22], with a subset of CpG sites more likely to be differentially methylated between unrelated individuals. We proposed that these CpG sites may be especially susceptible to environmentally-induced changes associated with placental disease [21]. In a follow-up investigation, we also observed a gestational age difference in DNA methylation profile in the placenta across the third trimester [23], while others have recently reported an increase in global DNA methylation levels between pre-term (28 weeks) and full term placenta (40 weeks) [24].

The aim of the current study was to build on recent knowledge obtained through genome-scale gene expression [5,6,25] and DNA methylation analysis [24,26] of the human placenta. In the current study, we used the Illumina Infinium HumanMethylation27 BeadChip platform to identify promoter regions subject to change throughout gestation. In addition we wanted to identify those that become increasingly variable between individual placentas over time, possibly in response to accumulated environmental exposures.

## Results

### Genome-scale DNA methylation analysis of first, second, and third trimester placenta

**Figure 1:**
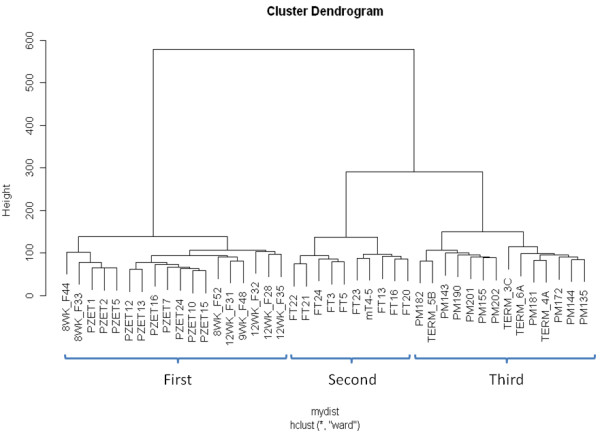
**Cluster dendrogram based on all autosomal Infinium probes distinguishes placentas of different gestational age**. Dendrogram showing the relationship between placental samples from three gestational ages based on DNA methylation levels (β-values) of all analysable Infinium probes. All samples clustered within their gestational age group, with no overlap between gestations, suggesting there are consistent genome-scale DNA methylation patterns associated with each gestational age. First trimester samples clustered away from second and third trimester samples, indicating that overall Infinium methylation patterns are more similar in second and third trimester compared to first trimester.

Genome-scale DNA methylation analysis of 18 first trimester (8-12 weeks), 10 second trimester (17-24 weeks) and 14 third trimester placenta (34-41 weeks) samples was performed using the Illumina Infinium HumanMethylation27 BeadChip (see Additional file 1; Data was deposited into the NCBI Gene Expression Omnibus, accession number: GSE31781). Following normalisation and data cleaning (see Methods), a total 26, 162 probes were available for subsequent analysis. Correlations (r^2^) of average methylation of probes between gestational ages ranged from 0.935 (first v third trimester) to 0.97 (second v third trimester; see Additional file 2). Unsupervised hierarchical clustering of all probes clearly delineates samples according to gestational age (Figure 1) with further evidence for a closer relationship between second and third trimester profile than with first trimester. Validation of methylation levels at 12 Infinium probes (representing 12 genes) in 9 placental samples (total data points: 49) using the Sequenom EpiTYPER platform confirmed the robust nature of the Infinium data (r^2 ^= 0.76) (see Additional file 3). The probes used for validation were chosen due to their association with genes with known or predicted important roles in regulating placental function (Additional file 4).

### Transition to normoxia is not associated with major changes in placental DNA methylation profile

Despite the overall interspersed pattern of clustering of first trimester samples of various gestations (8 - 12 weeks) (Figure 1), we tested to see whether any genomic regions undergo selective changes in methylation during the transition from early first trimester to late first/early second trimester. This is the period widely regarded as the time when placental intervillous space is flooded with oxygenated maternal blood. Only limited DNA methylation differences were observed between 8 and 12 week placentas (Additional file 5). A total of only 12 probes (3 hypomethylated and 9 hypermethylated at 12 weeks relative to 8 weeks) showed consistent methylation differences (Δβ) of 0.2 between the two gestational ages. This suggests that promoter DNA methylation plays a limited role in any physiological changes in the placenta that occur in response to altered oxygen status.

### Gestational age is associated with promoter methylation levels

**Figure 2:**
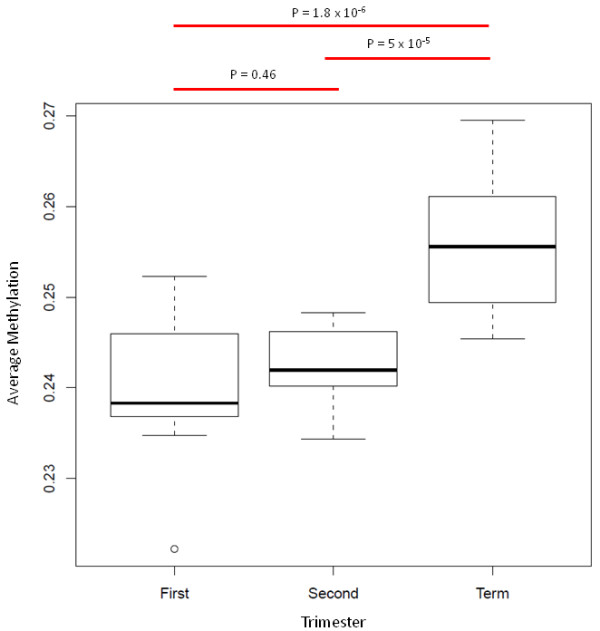
**Average methylation of all samples for first, second and third trimester**. Methylation Index (MI) was calculated for each sample by calculating the mean of all analysable Infinium β-values (26, 162 probes) for that sample. The MIs were then grouped by gestation and shown as box and whisker plots. First and second trimester placentas show a similar overall level of methylation (p = 0.46) with median MIs of 0.238 and 0.241, respectively. Third trimester samples show significantly elevated average MI values (median = 0.256) relative to both first and second trimester, indicating that there is a significant increase in methylation level from second to third trimester.

In order to gauge the effects of gestational age on the overall methylation level at gene promoter regions enriched on the Infinium HumanMethylation27 BeadChip, the mean methylation across all probes (Methylation Index - MI) was calculated for each sample (Figure 2). The mean MI for first, second and third trimester placenta was 0.240, 0.242 and 0.256, respectively. Thus, there is an overall increase in methylation between second and third trimesters (p = 5 × 10^-5^, student's t-test). No significant differences were detected between first and second trimesters (p = 0.46) (Figure 2). This is clearly apparent when the relative methylation levels for all probes are displayed in a scatterplot (Additional file 2). All 3 trimesters have the same proportion of probes with β-values between 0 and 0.2 (~63% of total probe number). However, as gestation progresses, there is an increase (from 13% to 17%) of highly methylated probes (β > 0.6) (Additional file 6).

**Figure 3:**
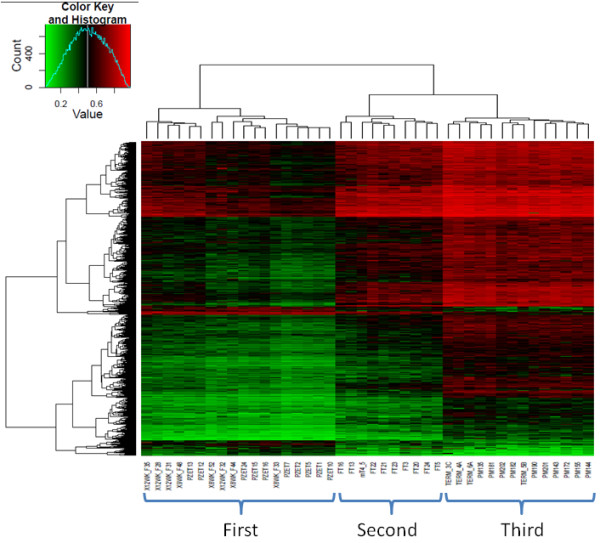
**Unsupervised clustering based on probes with Δβ > 0.2 between First and Third trimester**. HeatMap showing unsupervised clustering of all placenta samples (x-axis) based on 954 probes with a Δβ > 0.2 between First and Third trimester (y-axis). The majority of differentially methylated probes show higher methylation in third trimester (883 probes) compared to only 71 probes with lower methylation in third trimester. Second trimester placentas cluster as a separate group, and show a methylation profile that is an intermediate of first and third trimesters. Green corresponds to low methylation and Red to high methylation.

Absolute differences in mean methylation between first and second, first and third, and second and third trimesters were generally small, with only 149, 954 and 157 probes respectively, showing Δβ > 0.2 (Table 1). Further analysis showed that the 883 probes that increased in methylation from first to third trimester were predominantly those with intermediate methylation levels (average β range 0.3 - 0.5) in the first trimester (Additional file 7). Figure 3 shows a heat map with unsupervised clustering of samples based on the 954 probes showing a Δβ > 0.2 between 1^st ^trimester and term. Importantly, samples from all three trimesters showed distinct methylation patterns. More specifically, second trimester samples did not cluster with either the first trimester or term samples, suggesting a progressive change in methylation across gestation. Interestingly, none of the probes showed a 'fluctuating' pattern of methylation across gestation (i.e. showed a similar level of methylation in first and third trimesters, but hypo/hypermethylation in second trimester) based on our criteria of Δβ > 0.2, and only 103 probes showed this pattern with a Δβ > 0.1. This further suggests that most methylation changes occur in a progressive manner.

**Table 1:** Number of probes showing differential methylation between first, second and third trimester placental tissue.

Comparison	Differentially methylated probes (adj. p < 0.05)	Difference β ≥ 0.1 and adj. p < 0.05	Difference β ≥ 0.2 and adj. p < 0.05
**First v Second**	8, 240	411 ↓ 1, 077 ↑	12 ↓ 137 ↑

**First v Third**	8, 298	755 ↓ 2, 581 ↑	71 ↓ 883 ↑

**Second v Third**	7, 669	288 ↓ 1, 515 ↑	6 ↓ 151 ↑

↑: Increase in methylation; ↓: Decrease in methylation

Changes in cell composition, primarily a decrease in cytotrophoblasts, from first to third trimester could be one the major factors contributing to methylation differences between the two gestational time points. In order to test this, methylation levels between purified first trimester cytotrophoblasts and first and third trimester placenta, were examined using both Infinium and Sequenom EpiTYPER analyses (22 data points) (Additional file 8). Methylation levels in purified cytotrophoblasts were more similar to first trimester placental tissue (r^2 ^= 0.96 and 0.93) than third trimester (r^2 ^= 0.88 and 0.88) as calculated by Infinium and Sequenom EpiTYPER, respectively.

### Differentially methylated genes between first trimester, second trimester and term

Probes that showed differences of Δβ > 0.2 (approximating 20% differential methylation) between each of the three gestational ages, (Table 1), were analysed using Ingenuity Pathways Analysis (IPA). Enrichment of networks, pathways and gene functions was calculated using the Ingenuity Pathways software. Top enriched canonical pathways for genes differentially methylated between first and second trimesters (110 genes), first and third trimesters (654 genes) and between second and third trimesters (106 genes) are listed in Table 2. '*Communication between innate and adaptive immune cells' *was the most significantly enriched pathway, with at least 4 of the top 5 pathways in each comparison being immune-related.

**Table 2:** Top Canonical Pathways from IPA for probes showing differential methylation across gestation

First v Second trimester	p-value	# differentially methylated genes/# genes in the pathway
Communication between Innate and Adaptive Immune Cells	6.39E-05	6/109

Role of Cytokines in Mediating Communication between Immune Cells	1.53E-03	4/56

Altered T Cell and B Cell Signaling in Rheumatoid Arthritis	6E-03	4/92

Calcium Signaling	6.81E-03	6/204

Crosstalk between Dendritic Cells and Natural Killer Cells	1.04E-02	4/97

**Second v Third trimester**	**p-value**	**# differentially methylated genes/# genes in the pathway**

Systemic Lupus Erythematosus Signaling	4.72E-03	5/166

Crosstalk between Dendritic Cells and Natural Killer Cells	8.2E-03	4/97

Role of NFAT in Regulation of the Immune Response	2.05E-02	5/199

Agrin Interactions at Neuromuscular Junction	2.35E-02	3/69

Wnt/β-catenin Signaling	2.51E-02	5/172

**First v Third trimester**	**p-value**	**# differentially methylated genes/# genes in the pathway**

Communication between Innate and Adaptive Immune Cells	1.69E-07	17/109

Systemic Lupus Erythematosus Signaling	1.9E-06	21/166

Role of Cytokines in Mediating Communication between Immune Cells	1.43E-05	12/56

Altered T Cell and B Cell Signaling in Rheumatoid Arthritis	1.79E-04	13/92

Crosstalk between Dendritic Cells and Natural Killer Cells	2.61E-04	14/97

### Inter-placental methylation variation increases with gestational age

**Figure 4:**
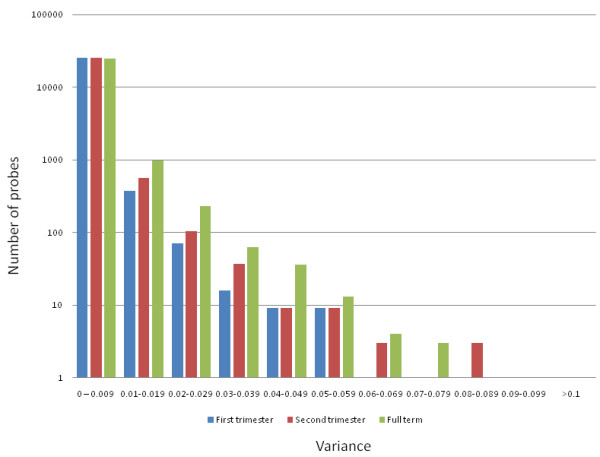
**Number of variable probes increases with gestation**. The number of probes with high inter-individual variation increases over gestation. Variance (*s^2^*) of each probe was calculated for each gestational age. The number of probes (y-axis; log_10 _scale) showing a particular level of variance (x-axis) is shown. Most probes (95% in third trimester to 98% in first trimester) show low variation (s2 < 0.009). However, there is an increase in the number of variable probes (*s^2 ^*> 0.02) in third trimester placentas, and to a lesser extent second trimester placentas, compared to first trimester.

DNA methylation may be modulated in part by environmental influences and may serve as a mediator between the environment and genome function (reviewed in [27]). We previously investigated the inter-individual variability of DNA methylation in the human placenta [21] and proposed that the highly variable methylation found in the placenta may be a consequence of cumulative response to the intrauterine environmental exposures [28]. To test this further, we calculated the inter-placental variance of all probes within each of the first, second and third trimester time points. While the vast majority of probes (95-98%) showed very little variation (s^2 ^< 0.009) at all time points, the number with an inter-placental variance of > 0.01 was increased in the third trimester relative to the earlier time points (Figure 4). More specifically, the third trimester group was enriched for probes with the highest variance (s^2 ^> 0.02), with 352 such probes compared to 106 (*χ^2 ^*= 133.3, p < 0.001) and 166 (*χ^2 ^*= 66.7, p < 0.001) in first and second trimester placentas respectively (Additional file 9). To facilitate the comparison of variation level between trimester groups, we defined sites with variance > 0.02 as highly variable in methylation.

Further analysis revealed that most of the gestational age associated variation was found in probes with intermediate methylation (0.2 < β < 0.6) rather than low (β < 0.2) or high (β > 0.6) methylation (Additional file 10; Additional file 9). This suggests that the increased variation observed between different full-term placentas is not necessarily a by-product of increasing methylation across gestation. In addition, the increasing methylation across gestation also suggests that the increasing variability was unlikely caused by a lack of maintenance of DNA methylation by DNMTs in the human placenta.

**Figure 5:**
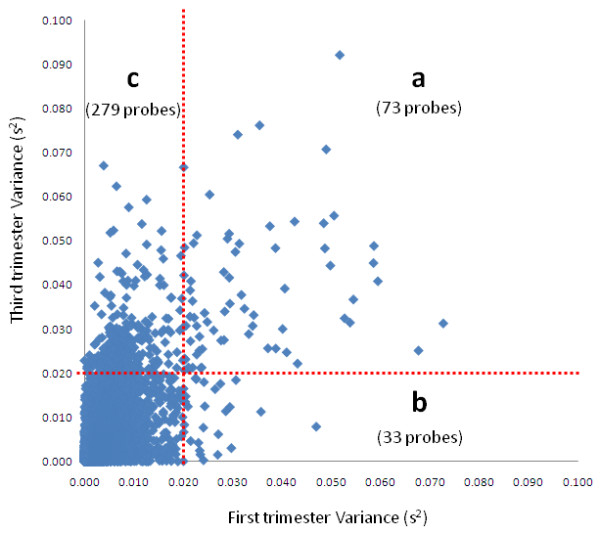
**Variance levels of probes in the third compared to the first trimester**. Scatter plot of probe variance (*s^2^*) at first trimester (x-axis) and third trimester (y-axis). Dots represent individual probes and the vertical red dotted line marks *s^2 ^*= 0.02 for first trimester, while the horizontal red dotted line marks *s^2 ^*= 0.02 for third trimester. Probes on the outside of the red line are deemed 'variable'. This analysis revealed that there are 73 probes (A) which are highly variable in both first and third trimester. Only 33 probes (B) were variable in first, but not third trimester, while 279 probes (C) were variable in third, but not first trimester. This analysis suggests that most of the variable probes become so throughout pregnancy, supporting the hypothesis that accumulating environmental factors contribute to inter-individual variation in DNA methylation, in term placenta.

While inter-individual variation of DNA methylation may in part reflect genetic polymorphisms [29], this source of variation would be anticipated to be represented equally across all 3 gestational ages. To investigate the inter-placental variation of methylation in more detail, variance level of probes for the third trimester was plotted against the first trimester (Figure 5). This revealed that the majority of the highly variable sites "gain" variability in the later gestations (area (c) in Figure 5). Numerically, the probes with the highest variance are found exclusively in the third trimester (223), while far fewer probes show high variation across all three gestations (47) (Additional file 11).

The top pathways for genes that show variable methylation at each gestastional age are listed in Table 3. The top 5 pathways in the third trimester include '*amino acid metabolism'*, while first and second trimester lists both include '*circadian rhythm signalling'*. This supports our hypothesis of an increasing accumulation of epigenetic variation in response to cumulative environmental exposures.

**Table 3:** Top Canonical Pathways for gene-associated probes that show variable methylation within each gestational age

Variable in First trimester	p-value	genes
Arachidonic Acid Metabolism	6.97E-04	*CBR1, CYP2E1, GPX7, PNPLA3, PTGS1*

Hepatic Fibrosis/Hepatic Stellate Cell Activation	1.36E-02	*CYP2E1, ECE1, FGFR1, MYL5*

Circadian Rhythm Signaling	1.91E-02	*GRIN3A, VIPR2*

Calcium Signaling	2.69E-02	*CHRNB4, GRIA4, GRIN3A, MYL5 *

Nitrogen Metabolism	3.54E-02	*PTPRG, VNN3*

**Variable in Second trimester**	**p-value**	***genes***

Circadian Rhythm Signaling	3.39E-03	*GRIN3A, VIPR2, PER1*

Glutathione Metabolism	1.62E-02	*GPX3, GPX7, GSTO1*

Arachidonic Acid Metabolism	1.86E-02	*CBR1, CYP2E1, GPX3, GPX7*

Sonic Hedgehog Signaling	3.7E-02	*DYRK1B, HKR1 *

Metabolism of Xenobiotics by Cytochrome P450	4.68E-02	*ADHFE1, CYP2E1, GSTO1*

**Variable in Third trimester**	**p-value**	***genes***

Glutamate Receptor Signaling	6.1E-03	*GRID2, GRIK2, GRIN3A, GRM6, SLC1A6*

Valine, Leucine and Isoleucine Degradation	1.13E-02	*ACADL, ALDH1A3, ELOVL2, IVD, OXCT1*

β-alanine Metabolism	2.07E-02	*ACADL, ALDH1A3, DPYS, IVD *

Butanoate Metabolism	3.74E-02	*ALDH1A3, ELOVL2, OXCT1, PDHA2 *

Tyrosine Metabolism	4.19E-02	*ADHFE1, ADLH1A3, ELOVL2, MGMT *

### CpG density and genomic context influence variability in placental methylation

In order to examine the relationship between CpG density and methylation status, probe locations were assigned to either CpG Island (CGI) or non-CpG Island (non-CGI) genomic regions, as annotated by Illumina. Using a chi square test, we found that probes showing increased methylation across gestation were enriched for non-CGI regions (χ^2 ^= 480.2, p < 0.001), while probes showing lower methylation in third compared to first trimester were not context dependent (χ^2 ^= 2.68, p = 0.14 (Additional file 12). We also examined the genomic context of the variable probes falling within the three categories shown in Figure 5(A-C). Probes with high variation in both first and third trimester (Additional file 12-D) are associated with CGIs (p < 0.05). Variable probes in the first, but not the third trimester are slightly enrichmed for CGI regions (p = 0.05), and those that were variable in third, but not first trimester show a strong association with CGIs (p < 0.001) (Additional file 12). We also looked at the relationship between the distance from transcription start site and DNA methylation. There was no association with distance from TSS and differential or variable methylation (data not shown).

### DNA methylation profile influences global gene expression in the placenta

**Figure 6:**
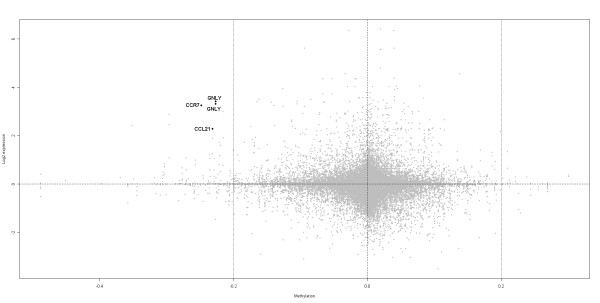
**Correlation between methylation and gene expression change between first and third trimester**. Methylation difference (Δβ) between first and third trimester (x-axis) was plotted against gene expression log fold change (y-axis) between first and third trimester. A positive change in log fold expression indicates higher expression in first trimester, while a positive change in methylation indicates higher methylation in the third trimester. Therefore, the top left panel includes genes which showed lower methylation and higher expression in first compared to third trimester. The three highlighted genes (CCR7, GNLY and CCL21) ranked highly in IPA analysis. Grey dots represent Infinium probes. Black dots represent specific genes of interest.

In order to assess the overall effect of DNA methylation on gene expression profile, methylation β-values levels for first, second and third trimester placental tissue were correlated with publicly available expression data for matched gestational age placenta [5,6]. Infinium probes were quartiled according to methylation level, with β value ranges of 0.01-0.06 (bottom 25% of probes), 0.06-0.15 (25-50%), 0.15-0.47 (50-75%) and 0.47-0.98 (top 25% of probes), and plotted against gene expression levels for linked genes data (Additional file 13). A general decrease in median expression level with increasing methylation level was observed for all three gestational ages. In particular, there was a distinct down regulation in median gene expression between the second (50%) to third quartiles (75%) associated with a change in methylation range from β < 0.15 to β > 0.15. In addition the range of expression was reduced in the higher methylation quartiles (Additional file 13). Using the same expression data sets, we plotted methylation change from first to third trimester against changes in gene expression across the same time points (Figure 6). While methylation change of less than 0.2 were generally not associated with changes in gene expression, several genes showed both higher methylation and lower expression in third trimester compared to first trimester. These included several immune-regulators ranked highly by IPA (*CCR7 *and *CCL21*) and one with known function in placental development (*GNLY*; Granulysin) (Figure 6). Additional file 14 lists additional genes that showed concordant differences in methylation and expression between first and third trimesters.

## Discussion

In this study we performed genome-wide DNA methylation analysis of gene promoter regions, using the Illumina Infinium HumanMethylation27 BeadChip, in placentas of different gestational ages (18 first trimester (8-12 weeks), 10 second trimester (17-23 weeks), and 14 third trimester samples (34-41 weeks)). We further validated the array data by targeting 12 CpG sites (covering 12 gene promoters) using the Sequenom EpiTYPER platform. The correlation of r^2 ^= 0.76 between the two platforms is comparable to that published for similar comparisons [30]. We found evidence for both a programmed change of methylation in gene promoters across gestation and an increase in variability of methylation as gestation progresses. We predict that this is directly related to the cumulative effect of intrauterine environmental exposure, however, the contribution of stochastic events to the observed variation cannot be discounted at this time. Further investigation is warranted to determine the effects of specific environmental exposures on DNA methylation patterns of the genes identified as variable in the present study.

Unsupervised clustering based on all Infinium probes (excluding those on the sex chromosomes) clearly separated all samples by gestational age, with first trimester samples clustering away from second and third trimester samples (Figure 1). This indicates that there are consistent, large scale changes in DNA methylation across gestation. There are several possible explanations for these temporal differences, one of which is the change in cell composition and differentiation from first to third trimester, especially the relative loss of cytotrophoblasts throughout gestatation, (85% of the total trophoblast population in first trimester but only 15% of the trophoblast volume at full term) [31]. In support of this playing at least a partial role in the observed change in methylation over time, we found a slightly higher correlation between the methylation profile of purified primary cytotrophoblasts with first trimester placental tissue (R^2 ^= 0.96) than with third trimester tissue (R^2 ^= 0.88; see Additional file 8). Similar data have recently been reported for second trimester placenta tissue which has been shown to be more similar to cytotrophoblasts than mesenchyme in terms of methylation profile [32]. However, the greater part of trophoblast volume at full term is syncytiotrophoblast, arising from the fusion of cytotrophoblast cells into a multinucleated layer. Thus, the observed trend of increasing methylation may equally be due to differentiation of the cytotrophoblast component, or alternatively, may even be due to other aspects of altered placental function known to occur as gestation progresses.

Despite the interspersed clustering of first trimester samples based on the overall Infinium methylation patterns, we were interested in assessing DNA methylation changes occurring at the transition from the first to the second trimester. This period of placental development is characterised by the loosening of trophoblast plugs and the associated rise in oxygen concentration from 2-3%, to 7-8% [33]. The rapid increase in oxygen levels can lead to the accumulation of reactive oxygen species (ROS), which might be linked to the reduction in trophoblast invasion and migration observed at about this time. Furthermore, it has been suggested that the inability of the placenta to adapt to this increase in oxygen concentration may lead to the development of preeclampsia [3]. In this study, we found only 12 CpG sites with Δβ > 0.2 between these time points, suggesting that DNA methylation changes are unlikely to play a major role in the physiological changes in placentation associated with transition from low oxygen to a normal oxygen environment. It must be noted however, that we do not have data on the level of vascularisation for our placental samples, so it is possible that flooding of the maternal blood may not have occurred at the time the 12 week tissue was collected.

The separation of the trophectoderm from the inner cell mass at the blastocyst stage occurs at a time when genome-wide DNA methylation levels are at their lowest [34]. The subsequent re-establishment of methylation marks occurs at a slower rate, and to a lesser extent, in the extra-embryonic lineage compared to somatic tissues [34]. This accounts for the low global DNA methylation in human placenta, which is more similar to human tumours, and is mostly due to hypomethylation of repetitive elements [35,36]. A recent study has reported a positive correlation between global DNA methylation levels (as measured by an ELISA-like assay with 5-methylcytosine antibody) and gestational age in the placenta [24], supporting our data for an increasing level of promoter-associated DNA methylation during gestation, particularly from second to third trimester (Figure 2). This accumulation of methylation was most apparent in genomic regions that showed an intermediate level of methylation in first trimester, suggesting that these genes are the most likely to be epigenetically regulated by DNA methylation. Furthermore, these CpG sites were more likely to be in lower CpG density regions (non-CGI) (see Additional file 12) suggesting that methylation levels of CpG sites within CpG Islands are more stable across gestation in this tissue. The higher promoter methylation at term could also reflect the end point of a continual process of re-methylation in the extra embryonic lineage from the blastocyst stage, at which point the genome is almost completely hypomethylated [34].

IPA analysis of genes showing differential methylation between first and third trimesters indicated that the most affected pathways were 'communication between innate and adaptive immune cells' and other '*immune-related*' pathways (Table 2). Genes in common between two or more of the top 5 immune-related pathways included immune regulators *CCR7*, *CD28*, *CSF2*, *IFNA17, IFNA2, IFNA21, IFNB1, IL1F7, IL2, IL3, IL5, LTA, TLR6, TLR9, TNF, TNFRSF13B *and *TNFSF13B*. This is in accordance with previous gene expression studies, which also showed substantial enrichment of immune regulators amongst the most differentially expressed genes in placenta at different gestational ages [5,6]. Several immune-regulators showed a strong correlation between methylation and expression change from first to third trimester, including Granulysin (GNLY), which has previously been implicated in spontaneous abortions [37] (Figure 6). Given the critical role of the placenta in modulating the maternal immune response to the developing pregnancy, and mounting evidence for a role of environmental exposures in controlling both immune-system development and epigenetic profile, it is not surprising that immune regulators are amongst the most variably methylated gene groups.

In addition to identifying CpG sites that consistently change over gestation, we were also interested in CpG sites that show inter-individual variability within each gestational age. Genes that show inter-individual differences in expression in placenta have previously been described by Sood et al. [25]. We have previously reported a subset of CpG sites showing variable methylation in term placenta between unrelated individuals, and suggested that these CpG sites may be more susceptible to change under adverse conditions [21]. We have now extended these findings by demonstrating that although ~95% of all probes showed very little variation (s^2 ^< 0.01) between individuals, the number of highly variable probes (s^2 ^> 0.02) increased with gestational age. Our data support a model whereby increasing variability in methylation profile between placentas arises in response to cumulative differences in environmental exposure rather than sequence polymorphisms (Figure 5). The most variable probes in the first trimester were associated with genes involved in, '*calcium signaling' *and '*nitrogen metabolism'*, while '*circadian rhythm signalling' *and '*arachidonic acid metabolism' *were enriched in both first and second trimester placenta (Table 3). The most enriched variable pathways at term included '*glutamate receptor signaling'*, '*valine, leucine and isoleucine degradation*', and '*β-alanine'*, '*butanoate'*, and '*tyrosine' *metabolism. The enrichment of pathways involved in metabolism further supports our hypothesis that the methylation variation is occurring in response to environmental influences, potentially as part of an 'adaptive' response of the developing pregnancy, thereby allowing changes in gene expression that may be more beneficial under altered environmental conditions [38]. The placental epigenome regulates placental gene expression and function, and any disruption in placental epigenetics (via either stochastic or environmental perturbation) has the potential to affect the developing fetus. Therefore it will be interesting in future to examine the potential for role of placental epigenome variation with the now widely accepted phenomenon of 'fetal programming' first described in the context of long term effects of intrauterine environment on offspring [39] and subsequently developed by Barker and colleagues in the early 1990s [40-42].

Finally, contrary to CpG sites that increase in methylation across gestation, variable CpG sites are more likely to be located within CpG islands (Additional file 12). This coupled with the finding that hypermethylated probes (β > 0.6) do not show higher variation compared to probes with low methylation levels (β < 0.2) (see Additional file 10) suggests that increased variation in late gestation is not a by-product of increasing methylation across gestation.

The direct role of DNA methylation in controlling placental global gene expression levels was examined by comparing our DNA methylation data with published gene expression data for placenta tissue of the same gestational age (Figure 6 and Additional files 13 and 14). A general trend of decreasing expression in response to increasing DNA methylation was found as expected. Further, the range of expression also decreased in the highly methylated probe group. These findings confirm that promoter DNA methylation influences global gene expression levels at all three gestational time points. However, the large range in gene expression in all four methylation quartiles, and the lack of strong correlation between methylation and expression change across gestation (Figure 6), support previous data that promoter DNA methylation is only one of the mechanisms controlling gene expression, with many other factors combining to regulate the expression of genes, including histone modifications, non-coding RNAs, and transcription factors.

## Conclusions

In the present study, we performed genome-scale DNA methylation analysis of human placenta at three distinct gestational ages, using the Infinium HumanMethylation27 array. This platform targets over 14, 000 genes, however, is limited to the 5' promoter region. Our findings support the hypothesis that DNA methylation levels in the human placenta are dynamic and change over gestation, possibly in response to changing cellular composition and/or cumulative environmental influences. The identification of pathways that are likely to be affected by the latter (i.e. those that show higher variation at full term) will provide valuable candidates for testing in studies examining placenta-associated adverse pregnancy outcomes.

## Methods

### Sample collection

Use of cells and DNA isolated from 8- and 12-week gestational tissue of normal pregnancies was approved by the Cambridgeshire Research Ethics committee (CREC 04/Q0108/23). Additional 1^st ^trimester (8-12 week gestation) and 2^nd ^trimester (17-24 week gestation) placental tissue was collected from elective abortions with the approval from the ethics committees of the University of British Columbia and the Children's & Women's Health Centre of British Columbia. Term placental tissue was collected as previously described [21,23,43]. Detailed information of the placental samples can be found in Additional file 1.

### DNA extraction

Tissue samples were incubated at 50°C overnight with shaking in DNA extraction buffer (100 mM NaCl, 10 mM Tris.HCl pH8, 25 mM EDTA, 0.5% (w/v) SDS), containing 200 μg/ml proteinase K. DNA was isolated by two rounds of phenol:chloroform extraction, followed by RNAse A treatment, precipitation in absolute ethanol containing 10% (v/v) sodium acetate (3 M, pH 5.2), and resuspended in 100 μl nuclease-free water (Ambion, Austin, TX, USA) or using salting out method followed by purification with Qiagen blood and tissue kit (Qiagen, Mississauga, ON, USA). DNA was stored at -20°C.

### Infinium DNA methylation analysis

Infinium arrays were hybridized and scanned as per manufacturer's instructions (Illumina, San Diego, USA). Individual probe β-values (range 0-1) were are approximate representations of the absolute methylation percentage of specific CpG sites within the sample population. The values were derived by comparing the ratio of intensities between the methylated and unmethylated alleles using the following formula:

$$\text{β}\;\text{value}=\frac{Max\left(Signal\;B,\;0\right)}{\left[Max\left(Signal\;A,\;0\right)\;+Max\left(Signal\;B,\;0\right)\right]}$$

Where Signal B is the array intensity value for the methylated allele and Signal A is the non-methylated allele. Samples were processed using the Bioconductor package *lumi*, which is specifically designed for Illumina data [44,45]. Samples were assessed for quality, color-adjusted to take into account the difference between batches, background-corrected and ssn normalized. Any probe within a sample with a detection p value of 0.05 or greater was excluded from further analysis. Probes on the × and Y chromosomes were removed from further analysis to eliminate sex-specific differences in methylation, leaving 26, 162 analysable probes. A batch correction effect was applied to the data in order to remove noise produced by processing samples at different times. Differentially methylated probes were defined as having a Δβ > 0.2 between groups, with an adjusted (Benjamini) p value < 0.1.

### Gene Ontology and Pathway analysis

Data sets were interrogated using the Ingenuity Pathways Analysis (IPA) application (Ingenuity^® ^Systems, Redwood City, CA; http://www.ingenuity.com). IPA was used to identify enriched canonical pathways, gene networks, functional classes, and toxicity lists (molecules involved in known toxicity processes).

### Locus-specific methylation analysis

Sequenom MassARRAY EpiTYPING was performed to validate Infinium methylation, as previously described [46]. Sequenom assays were designed to target specific Infinium probes. Genomic sequences for assay design were extracted from the UCSC genome browser http://www.genome.ucsc.edu/. Primer pairs for amplification were designed using EpiDesigner web tool http://www.epidesigner.com/. The primers are listed in Additional file 4. Amplification was performed after bisulfite conversion of genomic DNA with the MethylEasy Xceed bisulphite conversion kit (Human Genetic Signatures, North Ryde, Australia). Amplification conditions were 40 cycles: 95°C for 5 min, 56°C for 1 min 30 sec and 72°C for 1 min 30 sec, then 72°C for 7 min.

Raw data obtained from MassArray EpiTYPING was cleaned systematically using an R-script with the following three criteria (in order): (1) removal of samples that failed across 100% of CpG sites, (2) removal of CpG sites that failed (for example due to low or high mass, silent peaks, or low intensity readings) across more than 40% of samples, and (3) removal of samples that failed to generate data for more than 70% of CpG sites tested.

### Publicly available gene expression microarray data analysis

Gene expression data was downloaded from Gene Expression Omnibus (Barrett and Edgar, 2006, Sayers et al., 2009), (Database issue D885-D890). CEL files were downloaded from series GSE9984 [6] and GSE5999 [5] and processed with the Bio-conductor package 'gcrma' [47]. DNA methylation and gene expression data for the corresponding gestational age was compared as previously described [48]. The two expression matrices were merged based on probe ID as both were generated using the same array platform, the Affymetrix Human Genome U133A Array. Gene expression data was then linked with the methylation data according to gene name. Sample quartiles were produced from the methylation data. The expression values of the genes in each quartile were then plotted as box and whisker plots.

## Authors' contributions

BN analysed the data, participated in critical discussion, and wrote the draft manuscript. LG processed the array data and performed data analysis. RKCY, JMC, RS and WR designed the study, participated in critical discussion and wrote the manuscript. MSP set-up experiments and processed array data. AS and AM provided first trimester villi samples. All authors approved of the final manuscript.

## Supplementary Material

Additional file 1 **Summary of placental samples analysed in the study**. Click here for file

Additional file 2 **Correlations between average methylation in first, second and third trimesters**. Correlations between average methylation in (A) first and second trimesters, (B) second and third trimesters, and (C) first and third trimesters. This analysis revealed that first and third trimester methylation was most discordant, as expected, while second trimester placenta is more similar to third trimester placenta in terms of overall promoter DNA methylation. Furthermore, this figure visually shows the increase in methylation in third trimester compared to first (C). Click here for file

Additional file 3 **Correlation between Infinium and Sequenom methylation levels**. Correlation between Infinium HumanMethylation27 BeadChip and Sequenom EpiTYPER locus-specific methylation analysis. Methylation levels in 12 genes were measured using Sequenom MassARRAY Epityping targeting the same CpG sites interrogated on the Infinium BeadChip Arrays. Correlation between platforms was 0.76, supporting the use of the Infinium HumanMethylation27 BeadChip for profiling DNA methylation in this study. Genes interrogated are listed in Additional file 4. Click here for file

Additional file 4 **Sequenom EpiTYPER primer sequences**. Click here for file

Additional file 5 **Unsupervised clustering of first trimester placenta based on differentially methylated probes between 8 and 12 weeks gestation**. HeatMap showing unsupervised clustering of 8 and 12 week placenta samples based on 12 probes that showed a Δβ > 0.2 between 8 week and 12 week placenta. The 12 probes were associated with 11 genes, with two probes associated with the *BTG4 *gene. White corresponds to low methylation, and black to high methylation. Click here for file

Additional file 6 **Proportion of probes within a particular methylation level**. Pie Charts showing the proportion of probes within a particular methylation level for first, second and third trimester. The percentage of probes with a 'β < 0.02' is the same in all three gestational ages (63%), suggesting that probes with low methylation in first trimester remain low over placental development. Furthemore our data suggests that probes with an intermediate methylation in first trimester are the ones that increase over gestation, with a lower proportion of probes in the 'β = 0.2 - 0.6 group' in second (22%) and third (20%) compared to 1^st ^trimester (24%); and a higher proportion of probes in the β > 0.6 group in 2^nd ^(15%) and term (17%) placenta compared to 1^st ^trimester (13%). Click here for file

Additional file 7 **Average methylation level of probes that increase in methylation over gestation**. Box plot showing average methylation of (A) all probes (n = 26, 162) in first, second and third trimester placenta, and (B) probes that shown an increase in methylation from first to third trimester of β > 0.2 (n = 883). This analysis shows that probes with intermediate levels of methylation in first trimester are the ones that increase over time. On the other hand, probes with low methylation (β < 0.2) in the first trimester do not appear to increase in methylation in the third trimester placenta. Click here for file

Additional file 8 **Correlation between methylation levels in purified first trimester cytotrophoblasts and first and third trimester placenta**. Scatter plot showing correlation (r^2^) between first and third trimester placenta and purified first trimester cytotrophoblasts methylation, based on Infinium HumanMethylation27 BeadChip and Sequenom EpiTYPER analysis. The correlations between first trimester placenta and cytotrophoblasts were (A) 0.96 and (C) 0.93, and between third trimester placenta and cytotrophoblasts were (B) 0.88 and (D) 0.88, using Infinium and Sequenom platforms, respectively. This finding suggests that both first and third trimester placenta methylation levels are indicative of cytotrophoblast levels. The lower correlation in third trimester is likely due to both lower numbers of villous cytotrophoblasts and their differentiation into the syncytiotrophoblast layer. Click here for file

Additional file 9 **Number of probes showing variation at each gestational age**. Click here for file

Additional file 10 **Relationship between probe methylation level and variation**. Relationship between methylation level and inter-individual variation at each gestational age (*s^2^*). Probes were separated into three groups: (A) low methylation (β < 0.2), (B) intermediate methylation (0.2 < β > 0.6) and (C) high methylation (β > 0.6). The number of probes was plotted on the y-axis (in log_10 _scale) and the variance (*s^2^*) on the x-axis. Probes with an intermediate methylation level were most likely to show inter-individual variation (B), while probes with a high methylation level were least likely to show inter-individual variation. In fact, most of the probes with a variance of > 0.02 were from the intermediate methylation level (88/106 first trimester, 119/166 second trimester and 255/352 term), even though most of the Infinium probes (63%) are actually in the low methylation group. Click here for file

Additional file 11 **Number of probes showing variation at each gestational age**. Venn diagram of variable probes (*s^2 ^*> 0.02) in each gestational age. This analysis revealed that the vast majority of variable probes are only variable in third trimester, while 52 were only variable in second, and 21 only in first trimester. A total of 47 probes were variable across all gestational ages. Click here for file

Additional file 12 **Relationship between DNA methylation and genomic context**. Probes were separated into two groups based on their genomic location - CpG Island (CGI) or non-CpG Island (non-CGI). The expected frequency was based on the proportion of all analysable Infinium probes (A) within a CGI or outside a CGI (0.76 and 0.24, respectively). Probes that increased in methylation over gestation were predominantly in non-CGI regions (B), while probes that decreased in methylation over gestation showed the expected proportions. Furthermore, probes that showed inter-individual variation in (D) both first and third trimesters, (E) first trimester only, and (F) third trimester only, were predominantly associated with CGIs. This finding further suggests that probes that change over gestation are not the same as those that show inter-individual variation within each gestation. Click here for file

Additional file 13 **Relationship between DNA methylation and gene expression levels**. Relationship between DNA methylation and gene expression levels in first, second and third trimester placenta. Infinium HumanMethylation BeadChip probes were quartiled into 4 groups (0-25%, 25-50%, 50-75%, 75-100%) based on methylation level, with the same number of probes in each quartile. The quartiles for each gestational age were plotted on the x-axis with the corresponding gene expression values obtained from publically available first, second and third trimester data (y-axis). This analysis shows a decreasing median gene expression level with increasing DNA methylation, highlighting the functional relevance of DNA methylation in placenta at all three gestational ages. Click here for file

Additional file 14 **Correlation between methylation and expression change between first and third trimester with more genes highlighted**. Methylation difference (Δβ) between first and third trimester (x-axis) was plotted against gene expression log fold change (y-axis) between first and third trimester. A positive change in log fold expression indicates higher expression in first trimester, while a positive change in methylation indicates higher expression in third trimester. Highlighted genes are those that show a correlation between methylation and expression level. Grey dots represent Infinium probes, black dots represent most differentially methylated and expressed genes. Click here for file
