# Supplementary material for: Evidence for widespread changes in promoter methylation profile in human placenta in response to increasing gestational age and environmental/stochastic factors
Source: BMC Genomics. 2011 Oct 28;12:529. doi: 10.1186/1471-2164-12-529 (PMC3216976; doi:10.1186/1471-2164-12-529)
Supplement: Additional file 1 — Summary of placental samples analysed in the study. [file 1471-2164-12-529-S1.DOCX]

**Supplementary Table 2. Summary of placental samples analysed in the study**

| **TRIMESTER** | **Sample ID** | **SEX** | **GA (wks d)** |
| --- | --- | --- | --- |
| **1st** | PZET1 | F | 8 - 12 wks |
| **1st** | PZET2 | M | 8 - 12 wks |
| **1st** | PZET5 | M | 8 - 12 wks |
| **1st** | PZET7 | M | 8 - 12 wks |
| **1st** | PZET10 | M | 8 - 12 wks |
| **1st** | PZET12 | M | 8 - 12 wks |
| **1st** | PZET13 | F | 8 - 12 wks |
| **1st** | PZET15 | M | 8 - 12 wks |
| **1st** | PZET16 | M | 8 - 12 wks |
| **1st** | PZET24 | M | 8 - 12 wks |
| **1st** | 8WK_F44 | M | 8wks |
| **1st** | 8WK_F52 | M | 8 wks |
| **1st** | 8WK_F33 | M | 8 wks |
| **1st** | 9WK_F48 | M | 9 wks |
| **1st** | F28 | F | 12 wks |
| **1st** | F31 | M | 12 wks |
| **1st** | F32 | M | 12 wks |
| **1st** | F35 | F | 12 wks |
| **2nd** | FT3 | F | 19 wks 5d |
| **2nd** | FT5 | M | 23 wks 5d |
| **2nd** | mT4-5 | F | 20 wks 2d |
| **2nd** | FT13 | F | 17 wks |
| **2nd** | FT18 | M | 20 wks 3d |
| **2nd** | FT16 | F | 18 wks 2d |
| **2nd** | FT20 | F | 20 wks 4d |
| **2nd** | FT21 | M | 20 wks 1d |
| **2nd** | FT22 | M | 21 wks 6d |
| **2nd** | FT23 | M | 20 wks |
| **2nd** | FT24 | F | 22 wks |
| **Term** | PM135 | M | 39 wks |
| **Term** | PM143 | M | 39 wks 2d |
| **Term** | PM144 | F | 41 wks |
| **Term** | PM155 | F | 41 wks 4d |
| **Term** | PM172 | F | 40 wks 5d |
| **Term** | PM181 | M | 39 wks |
| **Term** | PM182 | F | 39 wks 6d |
| **Term** | PM190 | M | 39 wks 1d |
| **Term** | PM201 | M | 38 wks 4d |
| **Term** | PM202 | F | 39 wks 4d |
| **Term** | TERM_3C | M | >34 |
| **Term** | TERM_5B | F | 34 wks |
| **Term** | TERM_4A | F | 37 wks |
| **Term** | TERM_6A | M | >34 |
