# Supplementary figures and images for: Evidence for widespread changes in promoter methylation profile in human placenta in response to increasing gestational age and environmental/stochastic factors
Source: BMC Genomics. 2011 Oct 28;12:529. doi: 10.1186/1471-2164-12-529 (PMC3216976; doi:10.1186/1471-2164-12-529)

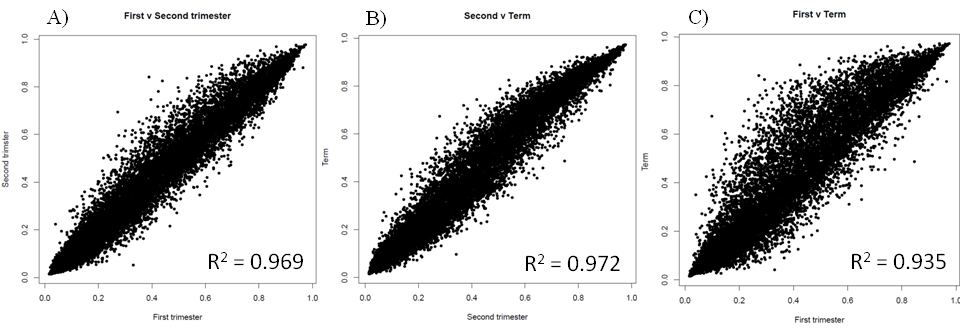

Supplement: Additional file 2 — Correlations between average methylation in first, second and third trimesters. Correlations between average methylation in (A) first and second trimesters, (B) second and third trimesters, and (C) first and third trimesters. This analysis revealed that first and third trimester methylation was most discordant, as expected, while second trimester placenta is more similar to third trimester placenta in terms of overall promoter DNA methylation. Furthermore, this figure visually shows the increase in methylation in third trimester compared to first (C). [file 1471-2164-12-529-S2.PNG]

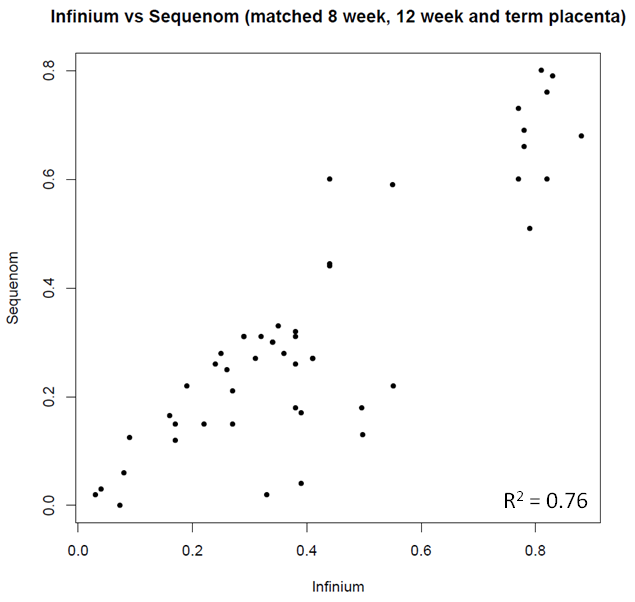

Supplement: Additional file 3 — Correlation between Infinium and Sequenom methylation levels. Correlation between Infinium HumanMethylation27 BeadChip and Sequenom EpiTYPER locus-specific methylation analysis. Methylation levels in 12 genes were measured using Sequenom MassARRAY Epityping targeting the same CpG sites interrogated on the Infinium BeadChip Arrays. Correlation between platforms was 0.76, supporting the use of the Infinium HumanMethylation27 BeadChip for profiling DNA methylation in this study. Genes interrogated are listed in Additional file 4. [file 1471-2164-12-529-S3.PNG]

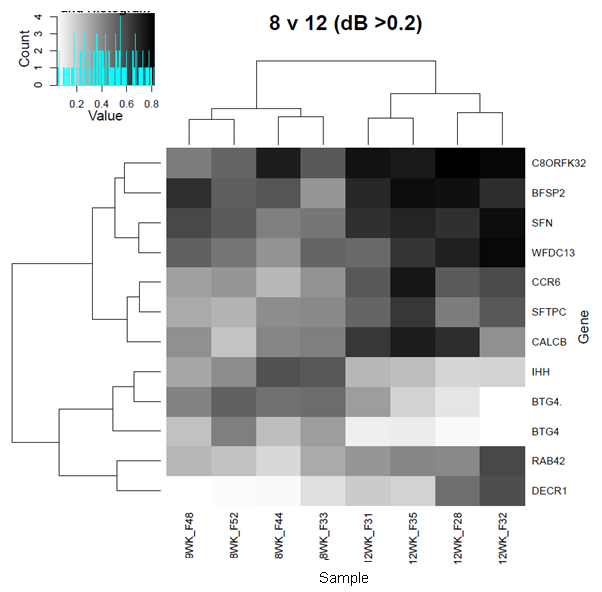

Supplement: Additional file 5 — Unsupervised clustering of first trimester placenta based on differentially methylated probes between 8 and 12 weeks gestation. HeatMap showing unsupervised clustering of 8 and 12 week placenta samples based on 12 probes that showed a Δβ > 0.2 between 8 week and 12 week placenta. The 12 probes were associated with 11 genes, with two probes associated with the BTG4 gene. White corresponds to low methylation, and black to high methylation. [file 1471-2164-12-529-S5.PNG]

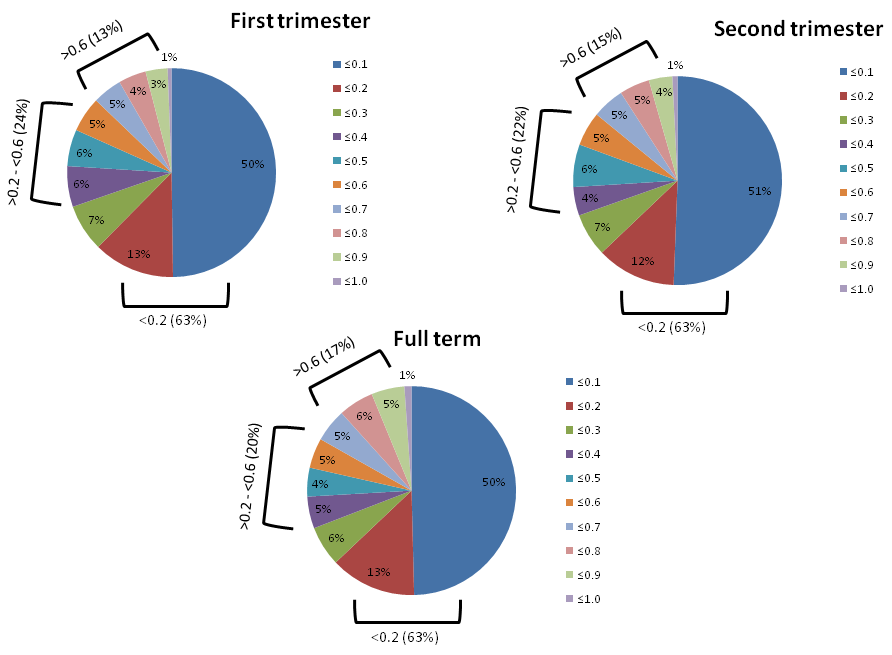

Supplement: Additional file 6 — Proportion of probes within a particular methylation level. Pie Charts showing the proportion of probes within a particular methylation level for first, second and third trimester. The percentage of probes with a 'β < 0.02' is the same in all three gestational ages (63%), suggesting that probes with low methylation in first trimester remain low over placental development. Furthemore our data suggests that probes with an intermediate methylation in first trimester are the ones that increase over gestation, with a lower proportion of probes in the 'β = 0.2 - 0.6 group' in second (22%) and third (20%) compared to 1st trimester (24%); and a higher proportion of probes in the β > 0.6 group in 2nd (15%) and term (17%) placenta compared to 1st trimester (13%). [file 1471-2164-12-529-S6.PNG]

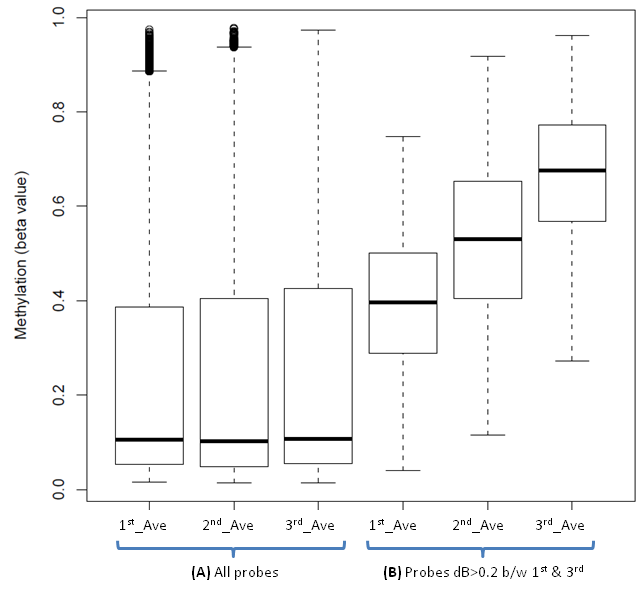

Supplement: Additional file 7 — Average methylation level of probes that increase in methylation over gestation. Box plot showing average methylation of (A) all probes (n = 26, 162) in first, second and third trimester placenta, and (B) probes that shown an increase in methylation from first to third trimester of β > 0.2 (n = 883). This analysis shows that probes with intermediate levels of methylation in first trimester are the ones that increase over time. On the other hand, probes with low methylation (β < 0.2) in the first trimester do not appear to increase in methylation in the third trimester placenta. [file 1471-2164-12-529-S7.PNG]

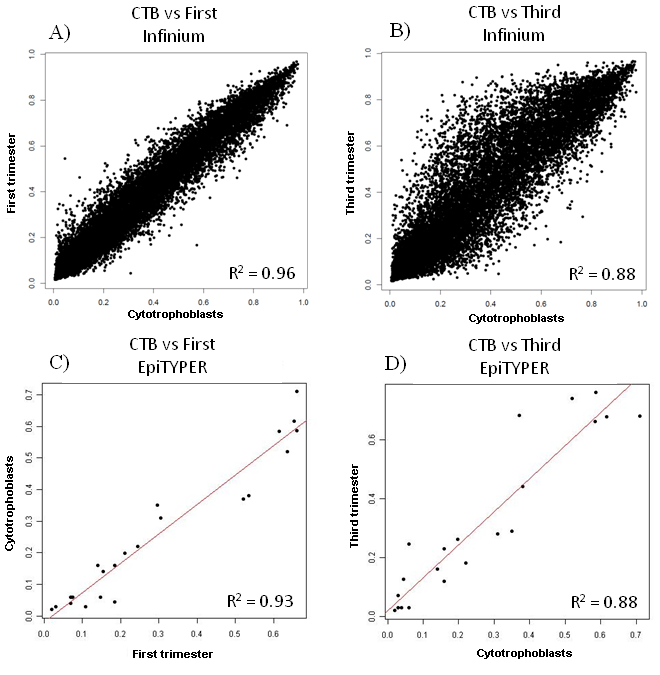

Supplement: Additional file 8 — Correlation between methylation levels in purified first trimester cytotrophoblasts and first and third trimester placenta. Scatter plot showing correlation (r2) between first and third trimester placenta and purified first trimester cytotrophoblasts methylation, based on Infinium HumanMethylation27 BeadChip and Sequenom EpiTYPER analysis. The correlations between first trimester placenta and cytotrophoblasts were (A) 0.96 and (C) 0.93, and between third trimester placenta and cytotrophoblasts were (B) 0.88 and (D) 0.88, using Infinium and Sequenom platforms, respectively. This finding suggests that both first and third trimester placenta methylation levels are indicative of cytotrophoblast levels. The lower correlation in third trimester is likely due to both lower numbers of villous cytotrophoblasts and their differentiation into the syncytiotrophoblast layer. [file 1471-2164-12-529-S8.PNG]

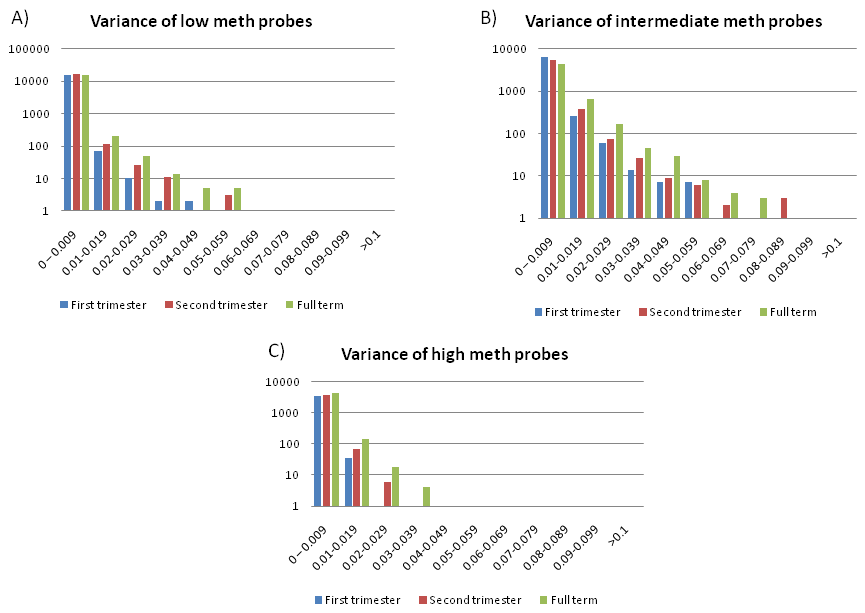

Supplement: Additional file 10 — Relationship between probe methylation level and variation. Relationship between methylation level and inter-individual variation at each gestational age (s2). Probes were separated into three groups: (A) low methylation (β < 0.2), (B) intermediate methylation (0.2 < β > 0.6) and (C) high methylation (β > 0.6). The number of probes was plotted on the y-axis (in log10 scale) and the variance (s2) on the x-axis. Probes with an intermediate methylation level were most likely to show inter-individual variation (B), while probes with a high methylation level were least likely to show inter-individual variation. In fact, most of the probes with a variance of > 0.02 were from the intermediate methylation level (88/106 first trimester, 119/166 second trimester and 255/352 term), even though most of the Infinium probes (63%) are actually in the low methylation group. [file 1471-2164-12-529-S10.PNG]

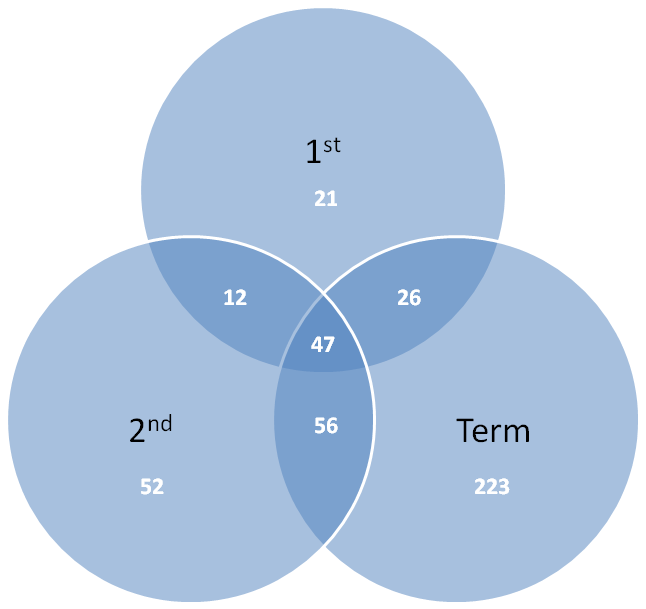

Supplement: Additional file 11 — Number of probes showing variation at each gestational age. Venn diagram of variable probes (s2 > 0.02) in each gestational age. This analysis revealed that the vast majority of variable probes are only variable in third trimester, while 52 were only variable in second, and 21 only in first trimester. A total of 47 probes were variable across all gestational ages. [file 1471-2164-12-529-S11.PNG]

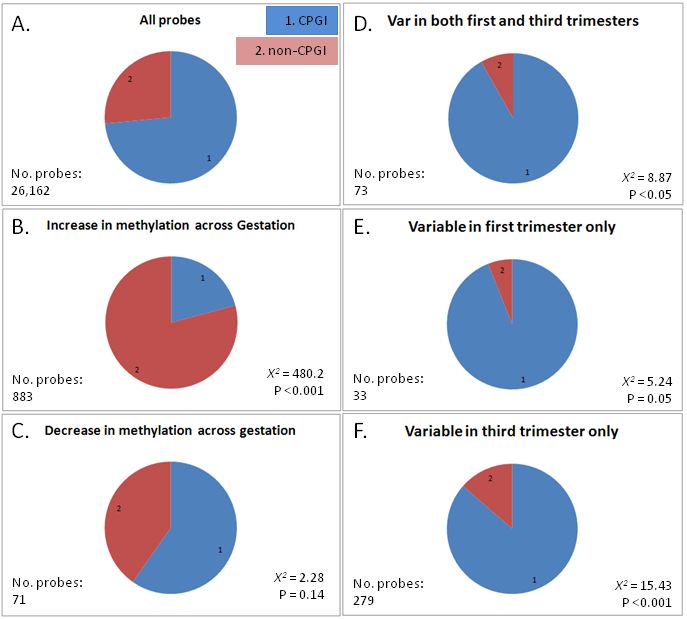

Supplement: Additional file 12 — Relationship between DNA methylation and genomic context. Probes were separated into two groups based on their genomic location - CpG Island (CGI) or non-CpG Island (non-CGI). The expected frequency was based on the proportion of all analysable Infinium probes (A) within a CGI or outside a CGI (0.76 and 0.24, respectively). Probes that increased in methylation over gestation were predominantly in non-CGI regions (B), while probes that decreased in methylation over gestation showed the expected proportions. Furthermore, probes that showed inter-individual variation in (D) both first and third trimesters, (E) first trimester only, and (F) third trimester only, were predominantly associated with CGIs. This finding further suggests that probes that change over gestation are not the same as those that show inter-individual variation within each gestation. [file 1471-2164-12-529-S12.PNG]

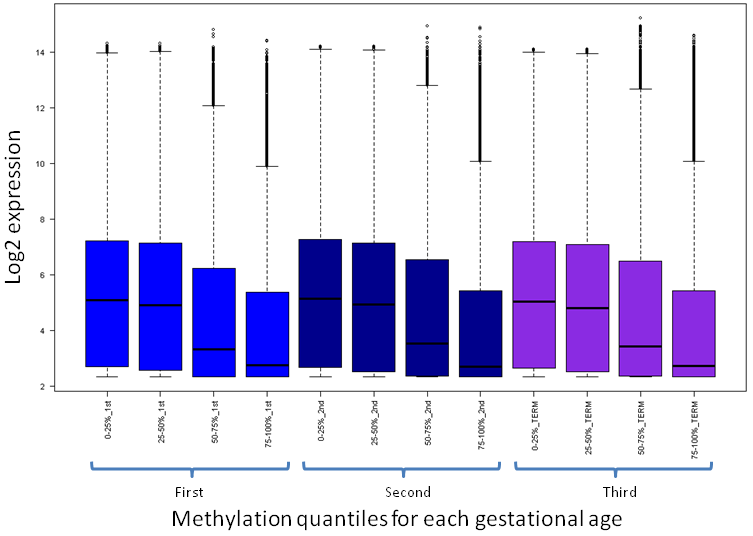

Supplement: Additional file 13 — Relationship between DNA methylation and gene expression levels. Relationship between DNA methylation and gene expression levels in first, second and third trimester placenta. Infinium HumanMethylation BeadChip probes were quartiled into 4 groups (0-25%, 25-50%, 50-75%, 75-100%) based on methylation level, with the same number of probes in each quartile. The quartiles for each gestational age were plotted on the x-axis with the corresponding gene expression values obtained from publically available first, second and third trimester data (y-axis). This analysis shows a decreasing median gene expression level with increasing DNA methylation, highlighting the functional relevance of DNA methylation in placenta at all three gestational ages. [file 1471-2164-12-529-S13.PNG]

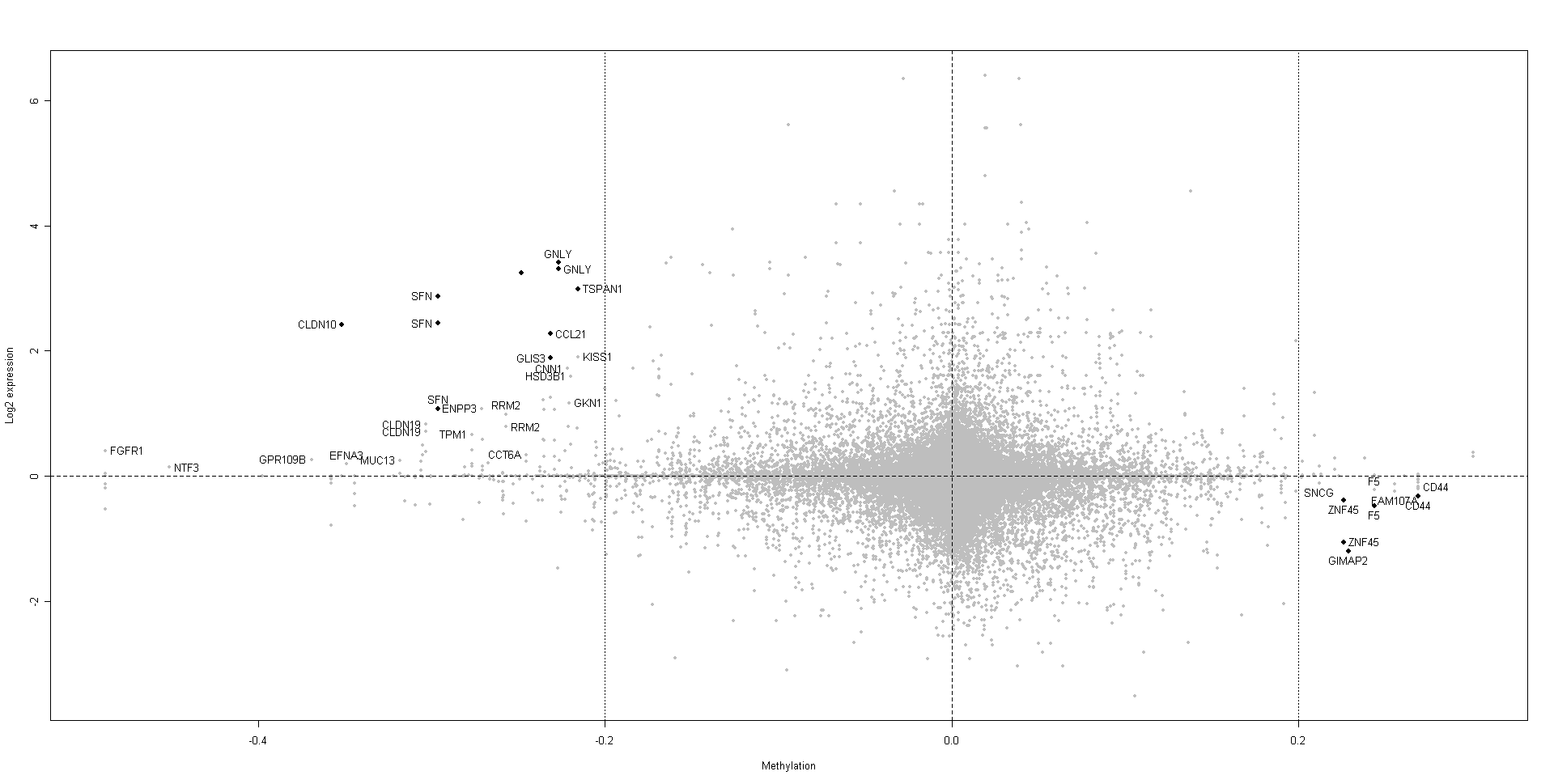

Supplement: Additional file 14 — Correlation between methylation and expression change between first and third trimester with more genes highlighted. Methylation difference (Δβ) between first and third trimester (x-axis) was plotted against gene expression log fold change (y-axis) between first and third trimester. A positive change in log fold expression indicates higher expression in first trimester, while a positive change in methylation indicates higher expression in third trimester. Highlighted genes are those that show a correlation between methylation and expression level. Grey dots represent Infinium probes, black dots represent most differentially methylated and expressed genes. [file 1471-2164-12-529-S14.PNG]
