# Supplementary material for: Evidence for widespread changes in promoter methylation profile in human placenta in response to increasing gestational age and environmental/stochastic factors
Source: BMC Genomics. 2011 Oct 28;12:529. doi: 10.1186/1471-2164-12-529 (PMC3216976; doi:10.1186/1471-2164-12-529)
Supplement: Additional file 4 — Sequenom EpiTYPER primer sequences. [file 1471-2164-12-529-S4.DOCX]

**Supplementary Table 1. Sequenom EpiTYPER primer sequences**

| **Gene Name** | **Primer Sequence** | **Product length (bp)** |
| --- | --- | --- |
| ***CMTM2*** | F 5’ aggaagagagTTTTAGGTGGTTTTAGGTTTGTAAGT 3’  R 5’ cagtaatacgactcactatagggagaaggctCCAACTTAAAAAAATTCCTAAAAATC 3’ | 390 |
| ***IL8RB*** | F 5’ aggaagagagGAGTTAGGATAGAGGGGAGAGAAAGT 3’  R 5’ cagtaatacgactcactatagggagaaggctAACAAAAAAAATACAAACATCCCAA 3’ | 195 |
| ***VAV1*** | F 5’ aggaagagagTAGGTAAAGAAGAGGAAGTGGTAGTAT 3’  R 5’ cagtaatacgactcactatagggagaaggctCAACTAACACAAAAAAACACCATCC 3’ | 260 |
| ***RASSF4*** | F 5’ aggaagagagTTGAAGTTTGGGTATTTGTGTTTTT 3’  R 5’ cagtaatacgactcactatagggagaaggctAACCTCTAAACTCTCTCCCCAACT 3’ | 253 |
| ***RASSF5*** | F 5’ aggaagagagGTTTTGTTTTTTTGTTGGGAGTAAG 3’  R 5’ cagtaatacgactcactatagggagaaggctAAAAAAAATACCCACCTCCCTC 3’ | 303 |
| ***TBC1D10C*** | F 5’ aggaagagagATAGGGGTAGGGTTTGGGGT 3’  R 5’ cagtaatacgactcactatagggagaaggctCCCAAAAAACTAAAATCATCCTACA 3’ | 293 |
| ***RARB*** | F 5’ aggaagagagTAGGGTTTGTATGTGTTTTTTTTGG 3’  R 5’ cagtaatacgactcactatagggagaaggctAACTCCCAAAATTCTCACAAAACTT 3’ | 387 |
| ***TNFRSF10A*** | F 5’ aggaagagagGTGTTGTTTTATGGAGGTAGGGAG 3’  R 5’ cagtaatacgactcactatagggagaaggctAAATTCAAAATTAACCAACAAAAACC 3’ | 318 |
| ***DUSP1*** | F 5’ aggaagagagAAAGGGGTTTTTTGTGTTTTTGTAT 3’  R 5’ cagtaatacgactcactatagggagaaggctAAAAAACATTACCCTAAACCTCTCC 3’ | 254 |
| ***GSTO1*** | F 5’ aggaagagagGAGTTAGTTAGGAGTTTGGGGAAGG 3’  R 5’ cagtaatacgactcactatagggagaaggctCCTAAATACCCACCTAATTCCCTTA 3’ | 347 |
| ***GPX7*** | F 5’ aggaagagagGTGAAATTGAGGTTTAGAGTYGGATAT 3’  R 5’ cagtaatacgactcactatagggagaaggctCCCAACAAACCCCRCCAAAA 3’ | 369 |
| ***APC*** | F 5’ aggaagagagTAATTTTTTTGTTTGTTGGGGATTG 3’  R 5’ cagtaatacgactcactatagggagaaggctATAACTCCAACACCTACCCCATTTC 3’ | 320 |
