# Supplementary material for: Evidence for widespread changes in promoter methylation profile in human placenta in response to increasing gestational age and environmental/stochastic factors
Source: BMC Genomics. 2011 Oct 28;12:529. doi: 10.1186/1471-2164-12-529 (PMC3216976; doi:10.1186/1471-2164-12-529)
Supplement: Additional file 9 — Number of probes showing variation at each gestational age. [file 1471-2164-12-529-S9.DOCX]

| **Gestation** | **Methylation level** | **0 – 0.009** | **0.01-0.019** | **0.02-0.029** | **0.03-0.039** | **0.04-0.049** | **0.05-0.059** | **0.06-0.069** | **0.07-0.079** | **0.08-0.089** | **0.09-0.099** | **>0.1** | **Totals** |
| --- | --- | --- | --- | --- | --- | --- | --- | --- | --- | --- | --- | --- | --- |
| **1^st^** | β <0.2 | 16028 | 72 | 10 | 2 | 2 | 1 | 1 | / | / | / | / | 16116 |
|  | β 0.2 - 0.6 | 6330 | 264 | 59 | 14 | 7 | 7 | / | 1 | / | / | / | 6682 |
|  | β >0.6 | 3328 | 34 | 1 | / | / | 1 | / | / | / | / | / | 3364 |
|  | **Total** | **25686** | **370** | **70** | **16** | **9** | **9** | **1** | **1** | **/** | **/** | **/** | **26162** |
| **2^nd^** | β <0.2 | 16298 | 118 | 26 | 11 | 0 | 3 | 1 | / | / | / | / | 16457 |
|  | β 0.2 - 0.6 | 5516 | 383 | 73 | 26 | 9 | 6 | 2 | / | 3 | / | / | 6018 |
|  | β >0.6 | 3614 | 67 | 6 | 0 | 0 | 0 | / | / | / | / | / | 3687 |
|  | **Total** | **25428** | **568** | **105** | **37** | **9** | **9** | **3** | **/** | **3** | **0** | **/** | **26162** |
| **3^rd^** | β <0.2 | 16180 | 211 | 50 | 14 | 5 | 5 | / | / | / | / | / | 16465 |
|  | β 0.2 - 0.6 | 4403 | 639 | 164 | 45 | 30 | 8 | 4 | 3 | / | 1 | / | 5297 |
|  | β >0.6 | 4238 | 139 | 18 | 4 | 1 | / | / | / | / | / | / | 4400 |
|  | **Total** | **24821** | **989** | **232** | **63** | **36** | **13** | **4** | **3** | **/** | **1** | **/** | **26162** |

**Supplementary Table 3.** Number of probes showing variation at each gestational age
